# Registered or unregistered? Levels and differentials in registration and certification of births in Ghana

**DOI:** 10.1186/s12914-018-0163-5

**Published:** 2018-06-13

**Authors:** Fidelia A. A. Dake, Kamil Fuseini

**Affiliations:** 1 Regional Institute for Population Studies, University of Ghana, P. O. Box LG 96, Legon, Accra, Ghana; 2 Population Council, P. O. Box CT 4906, Cantonment, Accra, Ghana

**Keywords:** Birth registration, Birth certificate, Ghana

## Abstract

**Background:**

The birth of a child is a vital event that needs to be registered but this is not always the case as an estimated 40 million births go unregistered annually. Birth registration safeguards the basic rights of children and gives them an identity, citizenship/nationality and legal protection against violence, abuse and human rights violations. It is therefore necessary that all births are registered and even more critical that the registration of a birth is followed by the issuance of a birth certificate. But sadly, birth registration in many African countries continues to remain below acceptable international standards and not all registered births are certified. This paper examined birth registration and certification in Ghana. Differentials in the characteristics of children and mothers of children whose births are registered and certified, children whose births are registered but not certified and children whose births are not registered were examined.

**Methods:**

This paper analysed data from the 2014 Ghana Demographic and Health Survey drawing on variables from the household and children’s data files. Descriptive analytical tools (frequencies, percentage and cross tabulations) and multinomial logistic regression analysis were used to examine differentials in birth registration status among an analytical sample of 3880 (weighted) children aged 0–4 years.

**Results:**

The birth of about every 1 in 4 (28.89%) children in Ghana have never been registered. Birth registration and certification was lowest among children born to young mothers (15–19 years), children whose mothers have no formal education, mothers who reside in rural areas and mothers in the poorest wealth quintile. Additionally, home births and births that were not assisted by a medical professional were observed to have the lowest proportion of registered and certified births. Furthermore, the birth of children who are less than a year old was significantly more likely not to be registered or issued with a birth certificate.

**Conclusion:**

Efforts aimed at improving birth registration and certification in Ghana need to target groups of children and mothers with low levels of registration and certification particularly children who are born at home, children born to young mothers and children whose mothers are poor and or reside in rural areas.

## Background

“Birth registration, the official recording of a child’s birth by the government, establishes the existence of the child under law and provides the foundation for safeguarding many of the child’s civil, political, economic, social and cultural rights” (United Nations Children’s Fund (UNICEF), 2016 [1]). From the foregoing, it is apparent that registering the birth of every child, most preferably at birth is not only a basic and fundamental human right but also that, birth registration has legal implications. Birth registration gives a child the right to a name, an identity and offers protection against rights violations [1–3]. But even though protecting the rights of children through birth registration has long been recognized as a key fundamental human right as enshrined in Article 7 of the Convention on the rights of the child and other international treaties [4, 5], universal registration of birth is far from being achieved. Globally, an estimated 230 million children under the age of five have never been registered [1, 4, 5], resulting in what some authors refer to as the “scandal of invisibility” [6]. More than half of the worlds’ unregistered children are in Asia [1], and sub-Saharan Africa is reported to be the region with the highest proportion of children who are not registered at birth with countries such as Tanzania and Zambia having the lowest birth registration rates in the region [7]. Birth registration among children under five stands at 38% in Eastern and Southern Africa with 44 million children unregistered. In West and Central Africa, the figure is slightly higher at 47% [5] but this still falls short of the target of universal coverage.

While it is necessary to ensure that all births are registered, the registration process is not complete if a birth certificate is not issued following registration. Without a birth certificate, the legal protection, identity and rights of the child is not guaranteed even though the birth of the child is registered. Furthermore, a birth certificate serves as proof of a child’s age and a potential means of protection against child labour, child marriage, child trafficking, sexual exploitation and forceful conscription into the armed forces [1]. With the proof of age backed by a birth certificate, children can potentially be protected from being arrested and treated as adults in the judicial system [1]. Additionally, birth certification helps to facilitate safe migration and tracing of unaccompanied and separated children. It is therefore of utmost importance that issuance of a birth certificate accompanies every birth that is registered. But, despite the countless benefits of possessing a birth certificate, an estimated 290 million children representing approximately about 45% of children under the age of five the world over do not have a birth certificate [1].

The importance of birth registration has become a topical development issue particularly in regions such as Southern Asia and sub-Sharan Africa where just about half of children under the age of five have been registered [1]. In Ghana, the registration of births has improved over the years. Data from the Multiple Indicator Cluster Surveys reveal that registration of births among children under 5 years has increased from 51% in 2006 to 63% in 2011 [8, 9]. In 2014, the Ghana Demographic and Health Survey reported that 70.5% of children under age five were registered with the civil authorities, with 55.8% of them having a birth certificate and 14.7% not having a birth certificate [10]. The increase in the level of birth registration came about as a result of interventions put in place by government institutions and some development partners. These interventions include mobile registration exercises where the Birth and Death Registry goes to remote and hard to reach communities to register children who were not registered at birth. Another similar intervention is the “My First Day at School” programme which involves registering children when they enrol in school.

In spite of these relatively high levels of birth registration, there is little empirical evidence on barriers and drivers of birth registration in Ghana and other developing country context [11]. Amo-Adjei and Annim (2015) [12] examined the socio-economic determinants of birth registration, but only looking at whether the birth of the child was registered or not. The present study goes beyond what was done by Amo-Adjei and Annim by examining differences in the characteristics of children whose births are registered with a birth certificate, children whose births are registered but do not have a birth certificate and children whose births are not registered at all. The remaining sections of the paper include a brief description of birth registration and certification in Ghana followed by the methodology, results, discussion and conclusion sections.

## Birth registration in Ghana

The laws of Ghana as mandated by the Registration of Births and Deaths Act of 1965 (Act 301) requires that all births (and deaths) that occur in Ghana are registered [13]. Birth registration in Ghana has seen a mixed pattern of increase and decline over the last couple of years. In the last decade, through the intervention of agencies such as UNICEF and Plan International and their partnership with government agencies such as the Births and Deaths Registry and the Ghana Health Service, registration of children under the age of five has increased [14].

In spite of this success story [12], an estimated 1.2 million Ghanaian children under the age of five are not registered in any official document [15].

Vital event registration in Ghana started in 1888 in the then Gold Coast [16]. However, the focus at that time was the registration of deaths of expatriate workers of the then colonial government. Ghana’s civil registration system has gone through a series of transformations since 1888 and the laws establishing civil registration in the country have also been amended several times [17]. The first law establishing civil registration in the then Gold Coast was known as the Cemeteries Ordinance of 1888 [16]. It was first amended in 1891 and became known as the Births, Deaths and Burials Ordinance in 1912 which was once again amended in 1926. The Births, Deaths and Burials Ordinance was again replaced with the Registration of Births and Deaths Act 301 of 1965. Act 301 of 1965 is still in use and it is the most current legislation for civil registration in Ghana [16].

The provisions for civil registration have also changed as the various ordinances/acts were amended. In 1888, the Cemeteries Ordinance was only concerned with the registration of deaths. Birth registration was later introduced after the 1912 amendment [17]. The 1965 amendment (Act 301 of 1965) provides for the registration of births, foetal deaths and deaths as well as provision for burial grounds. Currently, the agency in charge of civil registration in Ghana is the Births and Deaths Registry. The Registry was established by Act 301 of 1965 within the Ministry of Local Government and Rural Development [16]. Prior to this, the host ministry changed several times with the Ministry of Health serving as host at one point in time. The core mandate of the Births and Deaths Registry is to “provide accurate and reliable information on all births and deaths occurring within Ghana for socio-economic development of the country through their registration and certification” [16:4] of vital events.

The Births and Deaths Registry operates a centralised system with the national head office in Accra being the central co-ordination and administrative office [16]. There are regional offices in each of the ten administrative regions in the country. There are also registration offices at the district level but not all districts have an operational/functional registry. District Registration Officers submit all registration forms to the Regional Office on a monthly basis for further processing and onward transmission to the national head office for national data compilation [16]. Registration records are kept at all three levels to secure the information for development activities at all three levels. The requirements and characteristics for birth registration in Ghana as per the provisions of Act 301 of 1965 indicate that the onus lies on the family to notify births for registration. Thus the importance families attach to birth registration may influence whether or not they register the birth of their children [18, 19]. Additionally, the socio-demographic characteristics of families coupled with supply side factors such as accessibility and proximity to registration centres can influence registration of births. Thus in seeking to improve the level of birth registration in Ghana, it is important to investigate the socio-demographic characteristics of children and their parents/families that are associated with birth registration and certification. This paper thus examines differences in the characteristics of children whose births are registered with a birth certificate, children whose births are registered but do not have a birth certificate and children whose births are not registered at all.

## Methods

### Source of data

This study draws on data from the 2014 Ghana Demographic and Health Survey (GDHS). The GDHS is a nationally representative sample survey which has been conducted every 5 years since 1988. The most recent round of the survey (2014 GDHS) was, however, conducted 6 years after the 2008 GDHS. The GDHS serves a number of policy and programmatic purposes. Policy makers for instance, use these surveys to monitor progress in population and health indicators. The surveys are also used to inform policy decisions such as allocation of limited resources to health services. Respondents for the 2014 GDHS were selected using a two-stage sampling technique. The first stage of sampling involved the selection of a total of 427 clusters (sample points) consisting of Enumerations Areas (EAs) delineated for the 2010 Population and Housing Census. The second stage of sampling involved the systematic selection of 30 households from the selected EAs [10]. The 2014 GDHS collected data on various demographic and health indicators including maternal and child health, fertility and birth registration among others. This study used data from the birth registration module extracted from the household and children’s data files and the sample was restricted to the last or most recent child aged 0–4 years born to women aged 15–49 years in the last 5 years preceding the survey. This criterion was used to get at the most recent birth registration practice (s) and also to minimise recall bias in reporting the birth registration status of children, since it was not necessarily mothers who responded to the questions on birth registration in the household questionnaire.

### Variables

The dependent variable in this study is birth registration status. The dependent variable was generated from the question that asked if children aged 0–4 years on the household roster have a birth certificate: *Does (NAME) have a birth certificate*? Responses to this question included four categories of: (1) has certificate, (2) registered, (3) neither, and (4) don’t know. The “has certificate” category denotes children whose birth has been registered and who have a birth certificate while the “registered” category denotes children whose birth is reported to have been registered but do not have a birth certificate. The “neither” category represents those children whose birth has not been registered and who do not have a birth certificate. For the purpose of this study, the “don’t know” category was excluded from the analysis. The dependent variable; birth registration status therefore includes three categories of; (1) neither registered nor has a birth certificate, (2) registered without a birth certificate and (3) registered and has a birth certificate.

The independent variables considered in this study were grouped under four main categories; (1) child’s characteristics, which included age (0 = 0 years, 1 = 1 year, 2 = 2 years, 3 = 3 years, and 4 = 4 years) and sex (0 = male and 1 = female), (2) mothers characteristics, which included age (0 = 15–19 years, 1 = 20–24 years, 2 = 25–29 years, 3 = 30–34 years, 4 = 35–39 years, 5 = 40–44 years and 6 = 45–49 years), level of education (0 = no education, 1 = primary, 2 = middle/JSS, 3 = secondary/SHS, and 4 = higher), marital status (0 = never married, 1 = currently married and 2 = formerly married) and wealth status (0 = richest, 1 = poorest, 2 = poorer, 3 = middle and 4 = richer), (3) geographic location which included place of residence (0 = urban and 1 = rural) and region of residence (0 = Greater Accra, 1 = Western, 2 = Central, 3 = Volta, 4 = Eastern, 5 = Ashanti, 6 = Brong Ahafo, 7 = Northern, 8 = Upper East and 9 = Upper West) and (4) delivery characteristics which include place of delivery (0 = home, 1 = public health facility and 2 = private health facility) and assistance at delivery (0 = medical officer, 1 = Traditional Birth Attendant (TBA), 2 = other^1^ and 3 = no one).

### Methods of analysis

Descriptive statistical tools including frequencies and cross tabulations were used to examine the characteristics of children and their mothers by birth registration status. The dependent variable for this study is nominal with three categories, hence, a multinomial logistic regression model was used to examine the factors associated birth registration status.

## Results

### Characteristics of children and their mothers

Descriptive results in Table 1 show that 29% of births were neither registered nor had a certificate, 15% were registered without the issuance of a birth certificate and a little more than half (56%) of the births were registered with a birth certificate. Twenty-nine percent (29%) of the children were less than 1 year of age and slightly more than half (52%) were male. About a quarter (24%) of the mothers in the sample were aged 25–29 years, which was about the same proportion as those aged 30–34 years. Regarding the level of education of the mothers, while about 26% of mothers had no formal education, 5% had higher than secondary level of education. Majority of the mothers were married (85%) and more than half (54%) were residing in rural areas. The regional distribution shows close to 18% of the mothers living in the Ashanti region while the distribution by wealth quintiles shows 21% of the mothers belonging to the poorest wealth quintile. About two-thirds (66%) of the mothers delivered their most recent child in a public health facility and more than seven in ten (75%) of births were assisted by a medical professional.

**Table 1:** Socio-demographic characteristics of study sample

Variable	Number	Percent
Birth registration status		
Neither registered nor has birth certificate	1121	28.89
Registered (without birth certificate)	597	15.38
Has certificate	2162	55.73
Age of child		
0	1131	29.15
1	1054	27.17
2	785	20.23
3	530	13.67
4	380	9.79
Sex of child		
Male	2029	52.30
Female	1851	47.70
Age of mother		
15–19	169	4.35
20–24	640	16.50
25–29	944	24.33
30–34	926	23.87
35–39	732	18.87
40–44	359	9.25
45–49	109	2.82
Mother's level of education		
No education	1011	26.07
Primary	753	19.42
Middle/JSS	1544	39.80
Secondary/SHS	392	10.09
Higher	179	4.63
Mother's marital status		
Never married	331	8.53
Currently married	3293	84.88
Formerly married	256	6.59
Place of residence		
Urban	1788	46.07
Rural	2092	53.93
Region of residence		
Western	393	10.13
Central	421	10.84
Greater Accra	641	16.53
Volta	301	7.76
Eastern	371	9.57
Ashanti	681	17.56
Brong Ahafo	345	8.89
Northern	454	11.70
Upper East	168	4.32
Upper West	105	2.71
Wealth quintile		
Poorest	824	21.24
Poorer	790	20.37
Middle	760	19.58
Richer	769	19.81
Richest	737	18.99
Place of delivery		
Home	970	25.01
Public health facility	2566	66.14
Private health facility	343	8.85
Assistance at delivery		
Medical officer	2938	75.72
TBA	559	14.40
Other	266	6.87
No one	117	3.02
Total (weighted)	3880	100.00

Source: Generated from GDHS, 2014

### Variations in birth registration status by child and mothers’ characteristics, geographic location and delivery characteristics

The results in Table 2 show that the proportion of births registered with a birth certificate generally increases with age, from approximately 39% among children aged less than one to approximately 67% among children aged 4 years. This indicates that birth registration and certification tends to be done late rather than early as preferred. The results give indication that birth registration status is independent of the sex of a child as there was no significant association between the sex of children and their birth registration status. Further, the results show similar patterns in the distribution of male and female children in the various categories of birth registration status (Table 2).

**Table 2:** Association between child and mothers’ characteristics, geographic location and delivery characteristics by birth registration status

Variable (Chi-square test statistic)^*p*-value^	Neither registered nor has a birth certificate	Birth registered without a birth certificate	Registered and has a birth certificate
Age of child (210.64)***			
0	40.74	20.53	38.73
1	25.62	14.80	59.58
2	23.57	11.77	64.65
3	25.95	11.04	63.01
4	17.75	15.2	67.06
Sex of child (0.24) ns			
Male	28.75	15.65	55.60
Female	29.04	15.10	55.86
Age of mother (90.83)***			
15–19	53.16	15.09	31.75
20–24	34.22	17.87	47.91
25–29	29.04	14.29	56.67
30–34	24.91	14.84	60.25
35–39	24.41	15.87	59.72
40–44	26.59	13.61	59.79
45–49	30.09	17.94	51.97
Mother's level of education (152.20)***			
No education	33.52	20.14	46.35
Primary	37.95	13.94	48.1
Middle/JSS	25.04	14.94	60.02
Secondary/SHS	21.77	12.36	65.87
Higher	13.37	5.04	81.59
Mother's marital status (23.13)*			
Never married	39.55	12.41	48.04
Currently married	27.58	15.69	56.73
Formerly married	31.92	15.28	52.8
Place of residence (202.059)***			
Urban	20.76	11.45	67.78
Rural	35.83	18.74	45.43
Region (393.19)***			
Western	37.15	6.43	56.42
Central	19.82	28.35	51.84
Greater Accra	18.66	8.639	72.71
Volta	49.64	7.956	42.40
Eastern	36.98	8.64	54.38
Ashanti	19.28	15.26	65.46
Brong Ahafo	41.83	15.27	42.89
Northern	30.26	25.88	43.86
Upper East	29.02	20.38	50.60
Upper West	22.37	31.05	46.58
Wealth quintile (393.19)***			
Poorest	40.71	21.4	37.89
Poorer	39.43	17.14	43.42
Middle	30.3	14.62	55.08
Richer	19.91	13.41	66.67
Richest	12.27	9.61	78.12
Place of delivery (192.42)***			
Home	42.15	20.39	37.46
Public health facility	24.96	14.19	60.85
Private health facility	20.77	10.14	69.09
Assistance at delivery (218.53)***			
Medical officer	24.59	13.74	61.66
TBA	36.55	25.14	38.31
Other	50.54	15.14	34.32
No one	50.83	10.51	38.66
Total (%, weighted n)	28.89 (1121)	15.38 (597)	55.73 (2162)

*ns* not significant

*p < 0.05, ***p < 0.001

Regarding the characteristics of mothers and the registration status of their children, the results show that a higher proportion of children born to young mothers (15–19 years) were neither registered nor had a birth certificate (53.2%) whereas among women aged 30–44 years, about six out of ten of their most recent births were registered with a birth certificate. Births that were neither registered nor had birth certificates were more common among women with primary education (38%) and lowest among mothers with higher level of education (13%). Furthermore, the results show that the proportion of births registered with certificates increases with increasing level of education, from 46% among mothers with no education to 82% among mothers with higher level of education. Birth registration with certification was highest among currently married women (57%) and lowest among never married women (48%). On the other hand, the highest proportion of births that have not been registered nor issued a birth certificate was observed among never married women while the lowest proportion was observed among currently married women (Table 2).

The distribution by place of residence shows a higher proportion of births which were neither registered nor issued a certificate (36%), and births which were registered without the issuance of a certificate (19%) being common in rural areas while births that were registered with a birth certificate were higher in urban areas (68%). The regional variations showed the Volta region recording the highest proportion (50%) of births that were not registered nor issued a birth certificate whereas just about one-fifth of births in the Greater Accra (19%), Ashanti (19%), and Central (20%) regions were neither registered nor issued a certificate. The results with regards to household wealth status show that the proportion of births that were neither registered nor issued a certificate decreased with increasing wealth status from approximately 41% among the poorest to 12% among the richest.

And as is to be expected, a higher proportion of births that occurred at home were not registered nor issued a birth certificate. On the other hand, the highest proportion of births registered with the issuance of a birth certificate was observed for births occurring in private health facilities (69%), followed by public health facilities (61%) and home births (37%). Similarly, registration of births with the issuance of a birth certificate was highest among children whose birth was assisted by medical professionals (62%) compared to births that were assisted by TBAs (38%), other people (34%) or no one (39%).

### Factors associated with birth registration status

The results of the multivariate logistic regression analysis shown in Table 3 indicate that the risk of a child not being registered nor issued a birth certificate or being registered but not issued a birth certificate was highest for children who are less than a year old compared to children who are 4 years old. The results with regards to the sex of the child show a non-significant association with birth registration status as was also found in the bivariate analysis. Considering the characteristics of mothers, the results show that the chances that births to mothers aged between 20 and 49 years are neither registered nor issued a birth certificate is significantly lower compared to mothers aged 15–19 years. However, there was no significant association between mother’s age and the likelihood of the birth of their child being registered without the issuance of a certificate versus being registered with a birth certificate. Also, the mothers' level of education does not make a difference in whether a child is neither registered nor issued a birth certificate compared to the child being registered and issued a certificate. However, the relative risks of births being registered without a birth certificate relative to being registered with a birth certificate was significantly lower for mothers with higher levels of education (RRR = 0.37, *P* < 0.05) compared to mothers with no education.

**Table 3:** Predictors of child registration status

Variable	Birth neither registered nor issued a certificate	Birth registered without a certificate
	RRR [CI]	RRR [CI]
Age of child ^{4}^		
0	4.25 [3.02, 5.98]***	2.16 [1.43, 3.25]***
1	1.53 [1.09, 2.14]*	0.94 [0.60, 1.48]
2	1.29 [0.91, 1.83]	0.72 [0.46, 1.14]
3	1.67 [1.14, 2.44]**	0.75 [0.48, 1.17]
Sex of child ^{Male}^		
Female	0.98 [0.81, 1.18]	0.94 [0.76, 1.16]
Age of mother ^{15–19}^		
20–24	0.56 [0.33, 0.93]*	0.84 [0.35, 1.99]
25–29	0.53 [0.30, 0.92]*	0.67 [0.29, 1.57]
30–34	0.43 [0.25, 0.75]**	0.72 [0.29, 1.77]
35–39	0.44 [0.24, 0.80]**	0.79 [0.33, 1.89]
40–44	0.43 [0.23, 0.80]**	0.58 [0.22, 1.53]
45–49	0.50 [0.25, 1.04]^+^	0.79 [0.27, 2.35]
Mother's level of education ^{No education}^		
Primary	1.10 [0.82, 1.47]	1.03 [0.72, 1.46]
Middle/JSS	0.87 [0.67, 1.13]	1.07 [0.78, 1.47]
Secondary/SHS	0.95 [0.62, 1.46]	0.99 [0.62, 1.60]
Higher	0.70 [0.36, 1.34]	0.37 [0.17, 0.84]*
Mother's marital status ^{Never married}^		
Currently married	0.79 [0.59, 1.07]	1.03 [0.63, 1.67]
Formerly married	1.04 [0.66, 1.64]	1.22 [0.68, 2.17]
Place of residence ^{Urban}^		
Rural	1.12 [0.82, 1.52]	1.37 [0.91, 2.07]
Region ^{Greater Accra}^		
Western	1.22 [0.77, 1.93]	0.63 [0.32, 1.24]
Central	0.70 [0.44, 1.11]	2.81 [1.34, 5.87]**
Volta	1.72 [1.12, 2.63]*	0.98 [0.50, 1.92]
Eastern	1.10 [0.69, 1.76]	0.81 [0.38, 1.72]
Ashanti	0.68 [0.43, 1.06]^+^	1.45 [0.83, 2.55]
Brong Ahafo	1.53 [0.97, 2.40]^+^	1.95 [1.07, 3.58]*
Northern	0.49 [0.23, 1.02]^+^	1.94 [1.04, 3.62]*
Upper East	0.53 [0.31, 0.90]*	1.66 [0.85, 3.23]
Upper West	0.45 [0.22, 0.92]*	2.50 [1.21, 5.18]*
Wealth quintile ^{Richest}^		
Poorest	5.30 [3.09, 9.09]***	1.73 [0.92, 3.25]^+^
Poorer	3.44 [2.12, 5.59]***	1.44 [0.85, 2.46]
Middle	2.34 [1.51, 3.62]***	1.21 [0.72, 2.05]
Richer	1.60 [1.06, 2.41]*	1.16 [0.72, 1.86]
Place of delivery^{[Home}^		
Public health facility	0.65 [0.25, 1.73]	0.93 [0.22, 3.94]
Private health facility	0.58 [0.21, 1.63]	0.78 [0.15, 4.00]
Assistance at delivery ^{Medical Officer}^		
TBA	1.04 [0.41, 2.63]	1.66 [0.38, 7.33]
Other	1.42 [0.52, 3.93]	1.23 [0.27, 5.49]
No one	1.63 [0.60, 4.38]	0.84 [0.17, 4.01]

{} Reference Category, *RRR* Relative Risk Ratio

*N* = 3880, ^+^*p* < 0.1, *p < 0.05, **p < 0.01, ***p < 0.001

The regional results show that whereas children in the Volta region were more likely to neither be registered nor have a certificate compared to children in the Greater Accra region, births to mothers in Upper East and Upper West were less likely to neither be registered nor have a certificate. Additionally, births to mothers in Central, Brong Ahafo, Northern and Upper West regions were more likely to be registered without the issuance of a birth certificate compared to births in the Greater Accra region. With respect to wealth quintile, the risk of births not being registered nor having a birth certificate was observed to decrease with increasing wealth. However, there was no significant relationship between wealth quintile and births being registered without the issuance of a birth certificate. Additionally, place of residence did not show a statistically significant association with birth registration status. So was the case for mothers’ marital status and delivery characteristics (place of delivery and assistance at delivery).

## Discussion

This study examined the socio-demographic characteristics of children and their mothers that influence the birth registration status of children aged 0–4 years. The findings indicate that the sex of a child does not determine whether or not the child’s birth is registered as has been reported in earlier studies conducted in Ghana, South Africa and India [12, 20, 21]. This finding is however at variance with findings in Nigeria where the chances of birth registration was lower for female children compared to their male counterparts [22]. The findings with regards to age on the other hand indicate that children are less likely to be registered in the first year of life. Thus, birth registration and certification in Ghana appears to be late rather than the desired ideal of early registration. Similar findings have also been reported in Nigeria [22]. Some of the probable reasons for births being registered late rather than early is because a birth certificate becomes useful as the child grows older and accesses social services such as enrolling in school or acquiring a passport [12]. The tendency of registering a birth and acquiring a birth certificate later in life, however, negatively affects the chances of births being registered early as is the required standard practice.

The findings of the present study indicate that beyond the demographic characteristics of children, the socio-economic status of mothers’ plays an influential role on the birth registration status of their children. For instance, the chances of births being neither registered nor issued a birth certificate decreases with increasing mother’s age (although not consistent). Additionally, mothers with a higher level of education were significantly less likely to have their births registered without the issuance of birth certificates. Similarly, in countries such as Peru, Haiti, India, Brazil and Honduras, children born to illiterate parents have been found to be less likely to have a birth certificate compared to children born to literate parents [23]. Furthermore, studies from other developing countries have also found that mothers who are educated are more likely to have the birth of their children registered [24–27]. The level of education of mothers could influence birth registration status through a number of mechanisms. Firstly, education creates awareness of the importance of birth registration and thus probably explains why educated mothers are more likely to register the birth of their children. Secondly, being educated improves the socio-economic status of mothers and thus takes away the economic challenges associated with registration. As demonstrated in this study, poor mothers are less likely to have the birth of their children registered, suggesting that poverty is an obstacle to birth registration. Additionally, delivery outside a health facility and assisted delivery by persons who lack formal training increases the likelihood of a birth not being registered as was observed in the present study. Findings from other settings corroborate the findings of the present study. For instance studies in different parts of Nigeria reveal that delivery in a health facility was significantly associated with birth registration [26, 27] and in India, Mohanty and Gebremedhin (2018) found that institutional births were more likely to be registered.

In addition to the aforementioned socio-demographic variations in birth registration status, the results show spatial variations in birth registration status along a north south divide. The results show lower levels of birth registration in the southern part of Ghana compared to the northern part as has been found in other studies conducted in Ghana [12]. This north south divide is probably because of the concentration of interventions and research efforts in the Northern part of the country by numerous organizations including UNICEF, Plan International and the Community-based Health Planning Services (CHPS) Programme which has been operational in the northern regions for a much longer period of time than in other areas [12]. This concentration of research and interventions in the Northern part of Ghana may lead to higher levels of registration in the Northern part of Ghana compared to the Southern part. However, it is important that births that are registered in Northern Ghana are also issued with birth certificates. This is necessary because the results of this study show that births in the Northern region are more likely to be registered without the issuance of a birth certificate.

The findings of this study demonstrate that the characteristics of children and the socio-demographic characteristics of their parents/families particularly their mothers influence birth registration status. Unlike previous studies that focused on only birth registration in specific communities, the present study uses the most recent nationally representative data to examine birth registration as well as certification in Ghana. The findings are thus nationally representative and generalizable to the whole country. The study however has a few limitations that are worth mentioning. Firstly, there is the possibility that the proportion of births that are registered with a birth certificate could be over-reported as not all the certificates were seen by the interviewer. Secondly, the reporting of birth registration status may not be accurate as this information was asked at the household level and the required information may not have been provided by the child’s mother or parents. The findings of this study thus need to be interpreted in light of these potential limitations.

## Conclusion

The findings of this study indicate that while about every 1 in 2 children aged less than 5 years in Ghana are registered with a birth certificate, another 1 in 6 are registered but without a birth certificate. More importantly, the birth of about 1 in 4 children aged less than 5 years in Ghana have never been registered nor do they have a birth certificate. Furthermore, the findings demonstrate that there is a propensity for late rather than early registration. Additionally, while some births are reported to have been registered, these births were not issued with a birth certificate. It is important to educate the Ghanaian population on the importance of birth registration and more importantly that parents/guardians ensure that they obtain a birth certificate following the registration of a birth. Policy interventions aimed at improving birth registration and certification in Ghana need to target groups of children and mothers with low levels of registration and certification particularly children who are born at home, children born to young mothers and children whose mothers are poor and or reside in rural areas.

## Abbreviations

CHPS: Community-based Health Planning Services
GDHS: Ghana Demographic and Health Survey
TBA: Traditional Birth Attendant

## Footnotes

1 Other includes village health volunteer, traditional health practitioner, relative and other people.
